# A dual role of lipasin (betatrophin) in lipid metabolism and glucose homeostasis: consensus and controversy

**DOI:** 10.1186/s12933-014-0133-8

**Published:** 2014-09-13

**Authors:** Ren Zhang, Abdul B Abou-Samra

**Affiliations:** Center for Molecular Medicine and Genetics, School of Medicine, Wayne State University, 540 East Canfield Street, Detroit, MI 48201 USA; Department of Medicine, Hamad Medical Corporation, Doha, Qatar

**Keywords:** Angptl8, Betatrophin, C19orf80, Lipasin, RIFL

## Abstract

Metabolic syndrome includes glucose intolerance and dyslipidemia, both of which are strong risk factors for developing diabetes and atherosclerotic cardiovascular diseases. Recently, multiple groups independently studied a previously uncharacterized gene, officially named C19orf80 (human) and Gm6484 (mouse), but more commonly known as RIFL, Angptl8, betatrophin and lipasin. Both exciting and conflicting results have been obtained, and significant controversy is ongoing. Accumulating evidence from genome wide association studies and mouse genetic studies convincingly shows that lipasin is involved in lipid regulation. However, the mechanism of action, the identity of transcription factors mediating its nutritional regulation, circulating levels, and relationship among lipasin, Angptl3 and Angptl4, remain elusive. Betatrophin represents a promising drug target for replenishing β-cell mass, but current results have not been conclusive regarding its potency and specificity. Here, we summarize the consensus and controversy regarding functions of lipasin/betatrophin based on currently available evidence.

## Introduction

Metabolic syndrome represents common metabolic disorders that include glucose intolerance and dyslipidemia, and the prevalence of metabolic syndrome has increased dramatically in the past two decades [1]. Both glucose intolerance and dyslipidemia are strong risk factors for developing diabetes and atherosclerotic cardiovascular diseases [2,3]. Clinically, in addition to hyperglycemia and hyperinsulinemia, type 2 diabetic patients commonly suffer from disturbances in production and clearance of plasma lipoproteins, known as diabetic dyslipidemia, characterized by increased triacylglycerides (TAG), reduced high-density lipoprotein cholesterol (HDL-C) and postprandial lipemia [4-6].

In the postprandial state, plasma TAGs are mainly associated with chylomicrons (CMs) that are predominately synthesized in the intestine, while in the fasted state, TAGs are mainly associated with very low-density lipoprotein (VLDL) that is synthesized in the liver. Lipoprotein lipase (LPL) is critical in determining plasma TAG levels and the partitioning of fatty acids that are taken up by peripheral tissues [7,8]. In both mice and humans, loss-of-function mutations in LPL result in severe hypertriglyceridemia [9-11]. Similarly, loss-of-function mutations in GPIHBP1, which is required for proper targeting of LPL to the lumen of capillaries [12,13], also cause hypertriglyceridemia. Therefore, LPL expression and activity are tightly controlled to meet the needs of various tissues under different physiological and pathological conditions [8].

Recently, multiple groups independently studied a previously uncharacterized gene, officially named C19orf80 (human) and Gm6484 (mouse) according to the HUGO Gene Nomenclature Committee [14]. Both exciting and conflicting results have been obtained, and significant controversy is ongoing. In this review we aim to summarize the consensus and controversy using the currently available evidence regarding this gene.

The first controversy is that many different names have been proposed for this protein, including RIFL [15], lipasin [16-18], Angptl8 [19,20], betatrophin [21-24] and C19orf80 [25]. Here when mentioning an experiment or a finding, the name that the original authors used is adopted, so that all above names are used interchangeably. We define consensus as a result that has no published conflicting evidence or has been confirmed by independent studies.

### Consensus on the role of lipasin in lipid metabolism

#### Evidence from human genome-wide association studies

Multiple studies have identified ANGPTL8 sequence variations that are associated with lipid profiles in human genome-wide association studies (GWAS) [19,26-29]. The SNP rs2278426 represents a nucleotide transition (C vs. T, from CGG to TGG) that results in a non-synonymous amino acid change, from arginine (R) to tryptophan (W) at residue 59 (Table 1). The minor allele is T (W), and the minor allele frequency (MAF) is 15% in the 1000 Genome Project populations, which are mainly composed of whites [26]. The MAF is 26% in Hispanics, 18% in African Americans and 5% in European Americans [19].

**Table 1:** **ANGPTL8 SNPs that are associated with lipid levels**

**rsID**		**rs2278426**	**rs145464906**
Allele	Alleles	C/T	C/T
	Ancestral	C	C
	Minor allele	T	T
Position	Coordinates	Chr 19:11350488	Chr 19:11350874
	Strand	Forward strand	Forward strand
	Position in transcript	194	380
	Position in CDS	175	361
	Position in protein	59	121
Consequence	Consequence	Non-synonymous	Stop gained
	Codons	CGG to TGG	CAG to TAG
	Amino acids	R to W	Q to Stop
MAF		15.2% [26]	<1% [26]
		26%, Hispanics; 18%, AA; 5% EA [19]	0.1%, EUA; 0.01%, AFA [27]
Trait/Effect	HDL-C	Lower in AA (P = 2.1 × 10^−4^) and Hispanic (P = 0.025) [19]	10 mg/dl higher in EUA (P = 5.1 × 10^−11^) [27]
		14% lower in Mexicans (P = 3.4 × 10^−9^) [29]	
	LDL-C	15% lower in AA (P = 0.005) and Hispanics (P = 0.033) [19]	NS
	Triglycerides	NS	15% lower in EUA (P = 0.003) [27]

SNP information is based on dbSNP, release 138. AA, African American; AFA, African ancestry; CDS, coding sequence; Chr, chromosome; EA, European American; EUA, European ancestry; HDL-C, high-density lipoprotein cholesterol; LDL-C, low-density lipoprotein cholesterol; MAF, minor allele frequency; NS, non-significant; SNP, single nucleotide polymorphism.

Quagliarini *et al.* found that the 59W variant is associated with lower LDL-C in African Americans and Hispanics. In the Dallas Heart Study (DHS), African Americans and Hispanics with 59W homozygotes exhibited lower HDL-C than those with 59R homozygotes [19]. Consistently, in a study composed of 4361 Mexicans, Weissglas-Volkov *et al.* found that WW homozygotes had 14% lower HDL-C than RR homozygotes. African Americans in the DHS had 15% lower LDL-C in WW homozygotes than in RR homozygotes [29]. Of note, no association was found between R59W variant and triglyceride levels in both studies.

The SNP rs145464906 represents a nucleotide transition (C vs. T, from CAG to TAG) that results in a premature stop codon at residue 121, and therefore a truncated ANGPTL8 is generated by this SNP. The MAF of this SNP is extremely low, about 0.1% in European ancestry and 0.01% in African ancestry, and the carriers with European ancestry were 10 mg/dl higher in HDL-C and 15% lower in triglyceride levels [27].

The SNP rs737337 has also been found to be associated with HDL-C levels [28], and the SNP, although located in a region upstream of the ANGPTL8 transcription start site, represents a synonymous variant in the DOCK6 (dedicator of cytokinesis protein 6) gene. Therefore this SNP, although associated with lipid levels, may or may not be related to ANGPTL8 functions.

Taken together, multiple lines of evidence from GWAS suggest ANGPTL8 plays a role in lipid metabolism.

#### Evidence from mouse genetic studies

Mice that were deficient in Gm6484 were among the mouse knockout library created through collaboration between Genentech Inc. and Lexicon Pharmaceuticals Inc. This collaboration focused on genes that encoded secreted and trans-membrane proteins. A broad, unbiased phenotypic screen was performed on mouse lines in the library, and Gm6484-null mice clearly had lower triglyceride levels [30]. Wang *et al.* found that, based on an independent mouse line, Angptl8 knockout mice exhibited lower TAG levels in the fed state, but not in the fasted state. Furthermore, they showed that the knockout mice had a reduction in VLDL (very low density lipoprotein) secretion, an increase in LPL activity, and a markedly reduced uptake of fatty acids by white adipose tissue [20].

Overexpression of lipasin in mouse liver using adenovirus dramatically increased serum TAG levels [17]. Consistently, Quagliarini *et al.* found that adenovirus-mediated Angptl8 overexpression increased serum TAG levels, but in an Angptl3 dependent manner. Indeed, Angptl8 was co-immunoprecipitated with Angptl3 in mouse plasma and cultured hepatocytes, and promoted Angptl3 cleavage [19].

**Figure 1:**
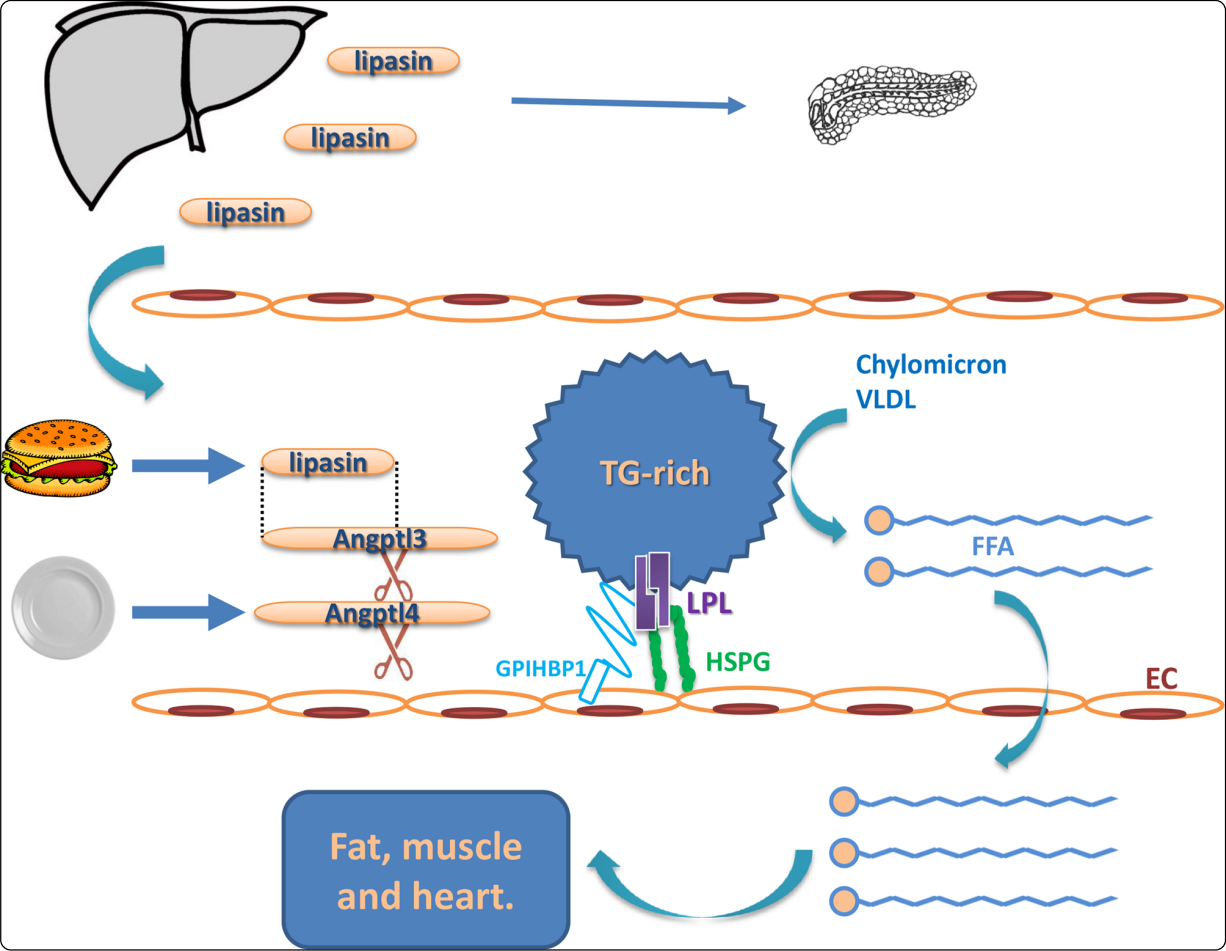
**Roles of lipasin (Angptl8) in regulating triglyceride metabolism and pancreatic beta-cell proliferation.** Lipasin is secreted from the liver into the circulation, and is involved in triglyceride metabolism and in promoting pancreatic β-cell proliferation. Active as a dimmer, LPL binds to both HSPG and GPIHPB1 on the surface of capillary microvascular endothelial cells. LPL hydrolyzes TAG in chylomicrons and VLDL, yielding FFAs, which are then taken up by peripheral tissues, including fat, muscle and heart. Both Angptl3 and Angptl4 need to be cleaved to release functional N-termini to inhibit LPL, disrupting dimer formation either reversibly or irreversibly, respectively. Lipasin likely inhibits LPL directly or indirectly by promoting Angptl3 cleavage. Food intake dramatically induces the expression of lipasin, whereas fasting induces Angptl4. Dotted lines denote homologous regions. Angptl3, angiopoietin-like 3; Angptl4, angiopoietin-like 4; EC, endothelial cell; GPIHBP1, glycosylphosphatidylinositol anchored high density lipoprotein binding protein 1; FFA, free fatty acid; HSPG, heparan sulfate proteoglycans; LPL, lipoprotein lipase; TAG, triglyceride; VLDL, very low-density lipoprotein.

Therefore, both loss- and gain-of-function studies in mice clearly suggest that lipasin is involved in triglyceride metabolism (Figure 1).

#### Expression pattern and transcriptional regulation

We found that in mice, lipasin is highly enriched in the liver, brown adipose tissue (BAT), and white adipose tissue (WAT), with liver being the tissue that has the highest expression, and in humans, it is predominantly in the liver among 48 tissues tested [17]. Ren *et al.* showed comparable expression of RIFL in mouse WAT, BAT and liver, and it is in human liver and WAT among the 4 tissues tested [15]. Quagliarini *et al.* showed comparable Angptl8 expression levels in liver, BAT and WAT, and also in the adrenal gland [19]. Because lipasin is sensitive to nutritional status, it is not surprising that there are discrepancies in expression patterns, especially in humans, when the nutritional status is not strictly controlled. Nevertheless, it is probably safe to conclude that lipasin is enriched in the liver and fat.

The mRNA levels of mouse lipasin in liver and fat are suppressed by fasting and elevated in mice with a high-fat diet treatment [17]. RIFL mRNA expression in fat and liver was found to be higher in *ob/ob* mice, which are obese due to the lack of leptin, and are highly induced by refeeding [15]. In mouse BAT, lipasin is induced by cold exposure [16]. C19orf80 is unregulated by T3 in the mouse liver [25]. In 3T3-L1 adipocytes, insulin increases, while cAMP decreases RIFL expression [15]. We found that that serum lipasin is increased 2 hours following a defined meal in humans [18]. Therefore, lipasin is clearly a liver-enriched nutritionally-regulated circulating factor.

#### Lipasin is a novel but atypical member of the ANGPTL protein family

The ANGPTL protein family contains 7 typical members, which are characterized by the presence of a coiled-coil domain at the N-terminus, a fibrinogen like domain at the C-terminus and a signal peptide for protein secretion [31]. N- and C-terminal domains of ANGPTLs have distinct functions. The N-terminal coiled-coil domain and the C-terminal fibrinogen-like domain of ANGPTL3 are involved in lipid regulation [32] and angiogenesis [33], respectively. Phylogenetic analysis using lipasin and N-terminal domains of the 7 ANGPTLs showed that lipasin and ANGPTL3 were most closely related, and shared a common ancestor with ANGPTL4 [16]. Quagliarini *et al.* suggested that ANGPTL8, which is in an intron of DOCK6, arose through gene duplication, because of a similar gene structure of ANGPTL3 and DOCK7 [19]. Of note, different from ANGPTL8 and ANGPTL3, ANGPTL4 is not in an intron of another gene. Lipasin, therefore, is a new but atypical member of the ANGPTL family, because it lacks the fibrinogen-like domain, but shares common ancestors with ANGPTL3 and ANGPTL4.

#### Role in adipogenesis and autophagy

RIFL is highly induced during adipocyte differentiation in 3T3-L1 cells, primary mouse and human pre-adipocytes. Indeed, knockdown using siRNA significantly suppressed adipocyte differentiation [15]. Tseng *et al.* found that C19orf80 is up-regulated in mouse liver by T3, a thyroid hormone that regulates liver lipid metabolism. Immunofluorescence analysis showed that C19orf80 is located around lipid droplets or within the lysosome-associated compartment. In hepatocyte cell lines, C19orf80 overexpression activates an autophagic response; conversely, shRNA mediated knockdown suppresses T3-activated autophagy and lipolysis. Therefore, C19orf80 is likely involved in an autophagic process that is activated by T3 in the liver [25].

### Controversy on the role of lipasin in lipid metabolism

Despite the consensus on the involvement of lipasin in lipid regulation, there has been inconsistent evidence regarding the mechanism in this process. The proposed mechanism is that lipasin inhibits LPL activity, and therefore in mice with lipasin overexpression, the higher triglyceride phenotype is likely due to the reduced triglyceride clearance by the inhibition of LPL activity [17]. Consistently, a recombinant lipasin inhibits LPL activity *in vitro. In vivo*, Angptl8 increases the cleavage of Angptl3 [19], releasing its N-terminal domain, which has been shown to inhibit LPL activity [32]. Indeed, in mice with Angptl8 deficiency, post heparin plasma LPL activity was increased [20].

However, contradictory to the above hypothesis, incorporation of free fatty acids (FFA) into adipose tissue was dramatically suppressed in the Angptl8 KO mice [20]. If lipasin inhibits LPL, we would expect increased circulating FFA levels in the Angptl8 KO mice because more triglycerides are hydrolyzed due to increased LPL activity, but the KO mice exhibit reduced FFA levels. Also, lipasin has been shown to be significantly induced by feeding and suppressed by fasting. In white adipose tissue, fasting reduces LPL activity, and lipasin expression is reduced as well. Therefore, it is not in line with an inhibitory role on LPL activity by lipasin, which is also reduced. Lipasin has been shown to be induced in brown fat by cold exposure. However, cold exposure increases LPL activity in brown fat, in which, however, lipasin expression is increased. Therefore, no satisfactory mechanism has been obtained to explain the relation between lipasin and LPL activity. Lipasin is predominately expressed in the liver [17]; LPL, however, is mostly expressed in fat and muscle [34]. The expression pattern difference seems to suggest lipasin to act in an endocrine manner. Nevertheless, lipasin is also highly induced in fat upon feeding. Therefore, whether lipasin acts in an endocrine manner, a paracrine manner, or both, is still a question.

**Figure 2:**
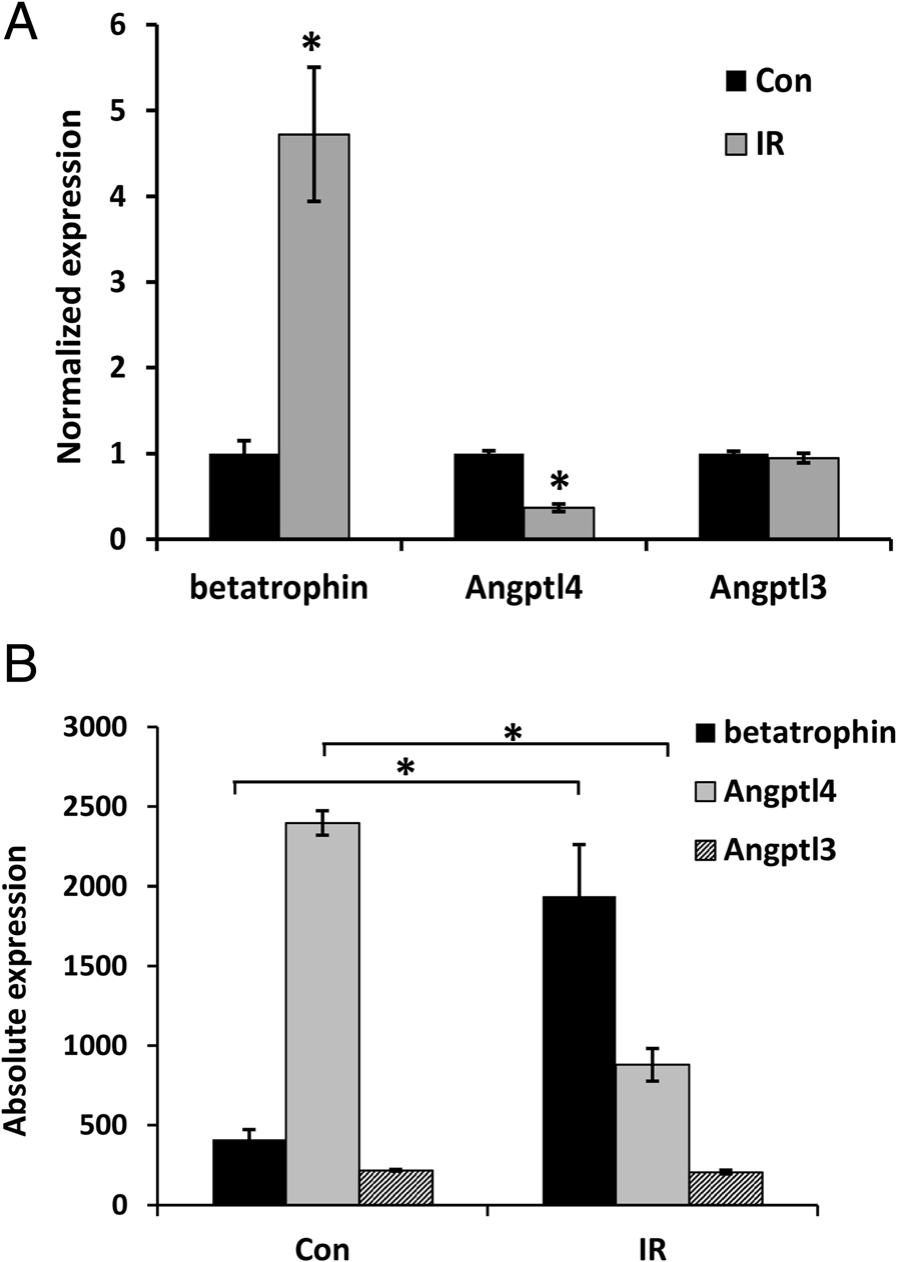
**Expression levels of betatrophin, Angptl4 and Angptl3 in the mouse model of insulin resistance induced by the insulin receptor antagonist S961. A)** Normalized and **B)** absolute expression levels of betatrophin, Angptl4 and Angptl3 in the liver of the mouse model of insulin resistance induced by the insulin receptor antagonist S961. The microarray data was obtained from the Gene Expression Omnibus with the accession number GSE45694. The gene symbol in the microarray dataset was Gm6484 (also known as Angptl8). Con, control; IR, insulin resistant.

We previously pointed out that lipasin and Angptl4 show opposite changes in expression by various stimuli, such as fasting, refeeding, obesity and cold exposure [35]. Yi *et al.* showed increased liver betatrophin in the insulin resistance mouse model [21]. Using the deposited microarray data from the GEO database [21], we compared the expression levels of betatrophin, Angptl4 and Angptl3. In this insulin resistance model, likewise, betatrophin was induced but Angptl4 was suppressed in the liver (Figure 2). It is possible that the 3 factors are coordinated in regulating lipid levels, however, the relationship among the 3 factors and how they are coordinated are far from clear.

In mice, TAG levels are increased by lipasin overexpression, and decreased by its deficiency, but cholesterol levels seem unaffected. In humans, however, most GWAS results show ANGPTL8 SNPs affect HDL-C or LDL-C levels, but the effect on triglycerides was very modest, compared to that on cholesterol (Table 1). The reasons for this discrepancy are unknown. Patients with type 2 diabetes are commonly associated with diabetic dyslipidemia [5,6], and altered LPL activity was shown to be involved in linking HDL-C metabolism and insulin resistance [36,37]. Because circulating lipasin levels are elevated in type 2 diabetes [18,23,38], it is possibly that lipasin plays a role in mediating lipid abnormality that is associated with insulin resistance.

RIFL is up-regulated during adipocyte differentiation, and PPARG was involved in the up-regulation [15]. In adipocytes, insulin stimulates expression of RIFL, which is down-regulated by cAMP [15]. C19orf80 is up-regulated by the thyroid hormone T3 [25]. We show that human lipasin and glucose levels are positively correlated, and there is a consensus ChREBP binding site in human lipasin promoter [18]. However, the exact transcription factors that coordinate lipasin transcription in response to food intake still remain elusive. It is important to identify the transcription factor, its *in vivo* binding sites, and the role it plays in response to feeding and insulin resistance, and to explain why the changes in lipasin and Angptl4 expressions are always opposite.

### Consensus on circulating factor(s) to promote pancreatic proliferation

Replenishing functional pancreatic β-cell mass represents a promising therapeutic strategy for treating both type 1 and type 2 diabetes. There is no doubt that human β-cells have the capacity to proliferate to significantly increase the mass for compensation, in response to physiological and pathological stimuli, such as pregnancy, high blood sugar, pancreatic injury and insulin resistance [39-43].

In mouse models, insulin resistance, induced by distinct methods, robustly promotes pancreatic β-cell proliferation. In a mouse line with insulin receptor deletion specifically in the liver (LIRKO), pancreatic β-cell mass is dramatically increased, along with phenotypes including insulin resistance, severe glucose intolerance, and a failure of insulin to suppress hepatic glucose production [43]. Yi *et al.* induced insulin resistance with an insulin receptor antagonist. In this model, in addition to dramatically increased insulin resistance, β-cell mass and proliferation are also dramatically increased [21].

Recently, El Ouaamari *et al.* showed that in the LIRKO mouse model, it is possible that liver-derived systemic factors contribute to the β-cell hyperplasia [44]. By performing parabiosis experiments, they first showed that circulating non-neuronal factors stimulate β-cell replication in the LIRKO mice. Next they showed that the serum from the LIRKO mice, when injected intra-peritoneally into the control mice, induced β-cell replication *in vivo*, indicating that circulating factors are responsible for the β-cell proliferation. Conditioned media from liver explant cultures or from primary hepatocytes showed β-cell stimulating effects, indicating that hepatocyte-derived circulating factors play a role in β-cell proliferation in the LIRKO mice [44]. Therefore, it is likely that one or more circulating factors can stimulate pancreatic β-cell proliferation. The question is what is (are) the circulating factor(s)?

### Controversy on betatrophin in β-cell proliferation

Yi *et al.* induced insulin resistance in mice by infusing an insulin receptor antagonist, S961, which is a 43 amino acid inhibitory peptide with high affinity and selectivity for the insulin receptor. This model of insulin resistance invoked dramatic pancreatic β-cell proliferation. Because S961 had no effect on β-cells *ex vivo*, a circulating factor was hypothesized to have this effect, and betatrophin expression was found to be induced in liver. Indeed, adenovirus mediated expression of betatrophin stimulated β-cell expansion and enhanced glucose clearance [21]. These results suggest betatrophin to be a liver-derived circulating factor that triggers compensatory β-cell proliferation upon insulin resistance.

Despite the initial excitement, the work triggered ongoing controversy, because subsequent work from other laboratories did not confirm a physiologic role for betatrophin in β-cell mass expansion. The first report showed that mice lacking Angptl8 had normal glucose homeostasis [20]. The second report showed that elevated hepatic betatrophin does not increase human β-cell replication in a transplant setting [45]. The two reports provide contradictory evidence for a physiological role of betatrophin in the mouse β-cell function or a pharmacological role for betatrophin on human β-cells; the conclusions, however, are far from conclusive.

The normal glucose tolerance of the betatrophin KO mice suggests that betatrophin is not required physiologically for the maintenance of the β-cell mass. However, a compensatory increase in homologous genes, such as Angptl3 and Angptl4, may have obscured the phenotype of the KO mice. The result that the human β-cells were completely unresponsive to betatrophin argues for lack of a physiological role for betatrophin in man. These experiments, however, as correctly pointed out by Dr. Andrew Stewart [46], lacked critical positive controls. For instance, it is possible that 1) the transplanted human β-cells were not capable of proliferation; 2) the overexpressed mouse betatrophin could not activate human receptors in the transplanted cells; and 3) despite the increase in betatrophin mRNA, the supposedly functional form, circulating betatrophin, was not increased. Therefore, the results would be more conclusive if a known human and/or mouse β-cell mitogen was overexpressed to show the transplanted human β-cells were responsive, and circulating betatrophin was measured to make sure it was increased. Taken together, it is hard to draw a conclusion on the role of betatrophin in β-cell function based on currently available evidence. Irisin is an exercise-induced hormone that stimulates thermogenesis through browning of adipocytes [47]. Irisin was found to promote betatrophin expression [48], and therefore an intriguing hypothesis of the p38-PGC-1α-irisin-betatrophin axis was proposed to connect these pathways [49].

### Controversy on circulating lipasin/betatrophin levels

To understand roles of betatrophin in human disease, there has been a surge in interest in examining circulating betatrophin levels in patients [18,22-24,38,50,51]. These studies show that betatrophin levels were altered in various physiologic conditions, such as the postprandial state [18], and pathological conditions, such as type 2 diabetes [18,23,38,50], type 1 diabetes [22], obesity [18,50], and were associated with metabolic parameters, such as BMI [18,50], glucose [18,38], insulin resistance [38,50], LDL-C [24], HDL-C and triglycerides [50].

However, results from these studies show a wide range of variations (Table 2). For instance, circulating levels of betatrophin in lean and non-diabetic subjects ranged from 0.3 ng/ml [22] to 45 ng/ml [50], and the levels were either increased [18,23,38] or decreased [50] in type 2 diabetes, either increased [18] or decreased [50] in obesity, either positively [18,38] or negatively [50] correlated with insulin, and either correlated with atherogenic lipid profiles [24] or with HDL-C [50] (Table 2). Indeed, we showed that for the same 30 human subjects, correlations between betatrophin and BMI can be either positive or negative based on ELISA kits that rely on antibodies recognizing the C-terminal or N-terminal betatrophin, respectively [51].

**Table 2:** **Circulating levels of lipasin/betatrophin and correlation with other parameters in different studies** ^**a**^

	**References**	[22]	[23]	[24]	[50]	[38]	[18]
Conditions	Lean non-diabetic	~300 (pg/ml)	639 (pg/ml)	1203 (pg/ml)^b^	45.1 (ng/ml)	296.6 (pg/ml)	2.19 (ng/ml)
	T1D	~doubled					
	T2D		893		13.5^c^	613.1	5.56
	Obesity				26.9		4.42
	Postprandial						30% increased
Correlation	BMI				*r* = −0.38		*r*_*s*_ = 0.49
	Cholesterol			*r* = 0.65			
	LDL-C			*r* = 0.61			
	HDL-C				*r =* 0.51		
	TAG				*r = −*0.36		
	Glucose					*r* = 0.34	*r*_*s*_ = 0.42
	HA1c		*r* = 0.48			*r* = 0.29	
	Insulin				*r* = −0.34	*r =* 0.28	*r*_*s*_ = 0.36
	Epitope^d^	N-Ter	N-Ter	N-Ter	NA	N-Ter	C-Ter

^a^The differences are statistically significant unless indicated otherwise.

^b^1643 pg/ml in another cohort.

^c^Obesity and T2D.

^d^Epitope refers to the epitopes of betatrophin antibodies in corresponding ELISA kits.

Positive correlation with age was noted in references [22,23,38]. Betatrophin levels were found to be higher in women (34.1 ng/mL) than in men (21.1 ng/mL) [50]. N-Ter and C-Ter, N-terminal and C-terminal antibodies were used in the ELISA kits manufactured by EIAAB and Phoenix, respectively. The ELISA kit used in [50] is manufactured by Cusabio (Hubei, China), and the antigen is recombinant betatrophin. HA1c, Hemoglobin A1c; HDL-C, high-density lipoprotein cholesterol; LDL-C, low-density lipoprotein cholesterol; T1D, type 1 diabetes; T2D, type 2 diabetes; TAG, triacylglyceride.

To resolve these discrepancies, we proposed that different betatrophin species are measured by ELISA kits that rely on either the N- or the C-terminal antibodies [51]. When human sera were analyzed by Western blot, the C-terminal antibody recognized both full-length protein (band position corresponding to 22kD) and C-terminal fragments (band position corresponding to small molecular weight) [52]. The small fragments were identified as amino acids 118–198 and 133–198 by HPLC and MALDI-TOF, and therefore, betatrophin is likely cleaved *in vivo* to release the C-terminal fragments, while the N-terminal fragments are degraded [52]. Therefore, the N-terminal kit likely measures full-length betatrophin and the C-terminal kit measures total betatrophin, including both full-length protein and the C-terminal fragments. Indeed, the betatrophin levels in lean and non-diabetic subjects determined by the C-terminal kit are higher than those determined by the N-terminal kit (Table 2).

There are at least two alternative explanations. The first is alternative splicing of the betatrophin transcript. Indeed, 2 additional transcripts are reported in the Ensemble database, in addition to the full length transcript ENSP00000252453 which encodes 198 residues: isoforms ENSP00000464941 and ENSP00000465378 encode 99 and 58 residues, respectively (lacking 99 and 140 N-terminal amino acid residues, respectively). However, the finding that only full-length transcript was detected in human liver [19] does not support the alternative splicing hypothesis.

The discrepancies could also be due to sample degradation. If the C-terminal fragments are more stable than the N-terminal ones, the C-terminal fragments accumulate in the circulation, resulting in higher total betatrophin. However, because of the presence of proteinase cleavage sites and the corresponding C-terminal fragments identified by mass spec *in vivo* [52], the explanation of proteolytic regulation is more likely. Regardless of the explanation, caution will have to be exercised in interpreting betatrophin ELISA results by considering which antibodies are used, and which betatrophin species is measured.

## Conclusion

Accumulating evidence from GWAS and mouse genetic studies convincingly shows that lipasin/Angptl8/RIFL is involved in lipid regulation. However, the mechanism of action, discrepancy between human and mouse studies and the relationship among lipasin, Angptl3 and Angptl4, remain elusive. Betatrophin represents a promising drug target in replenishing β-cell mass, but further evidence is needed to support this result.

## Abbreviations

Angptl: Angiopoietin-like protein
BAT: Brown adipose tissue
BMI: Body mass index
cAMP: Cyclic adenosine monophosphate
ChREBP: Carbohydrate-responsive element-binding protein
CM: Chylomicrons
C-terminus: Carboxyl-terminus
DHS: Dallas Heart Study
Dock6: Dedicator of cytokinesis 6
ELISA: The enzyme-linked immunosorbent assay
FFA: Free fatty acids
GEO: Gene Expression Omnibus
GPIHBP1: Glycosylphosphatidylinositol anchored high density lipoprotein binding protein 1
GWAS: Genome wide association study
HDL-C: High-density lipoprotein cholesterol
HPLC: High-performance liquid chromatography
KO: Knockout
LDL-C: Low-density lipoprotein cholesterol
LIRKO: Liver-specific insulin receptor knockout
LPL: Lipoprotein lipase
N-terminus: Amino-terminus
MAF: Minor allele frequency
MALDI-TOF: Matrix assisted laser desorption ionization time-of-flight
PAGE: Polyacrylamide gel electrophoresis
PGC-1α: Peroxisome proliferator-activated receptor gamma coactivator 1-alpha
Poly(A): Polyadenylation
RIFL: Refeeding induced in fat and liver
shRNA: Short hairpin RNA
SNP: Single-nucleotide polymorphism
T3: Triiodothyronine
TAG: Triacylglyceride
VLDL: Very low-density lipoprotein
WAT: White adipose tissue
